# A global ocean dissolved organic phosphorus concentration database (DOPv2021)

**DOI:** 10.1038/s41597-022-01873-7

**Published:** 2022-12-16

**Authors:** Zhou Liang, Kelly McCabe, Sarah E. Fawcett, Heather J. Forrer, Fuminori Hashihama, Catherine Jeandel, Dario Marconi, Hélène Planquette, Mak A. Saito, Jill A. Sohm, Rachel K. Thomas, Robert T. Letscher, Angela N. Knapp

**Affiliations:** 1 Department of Earth, Ocean, and Atmospheric Science, Florida State University, Tallahassee, FL USA; 2 Copperative Institute for Great Lakes Research (CIGLR), School for Environment and Sustainability, University of Michigan, Ann Arbor, MI USA; 3 Department of Oceanography, Faculty of Science, University of Cape Town, Cape Town, South Africa; 4 Marine and Antarctic Research centre for Innovation and Sustainability (MARIS), University of Cape Town, Cape Town, South Africa; 5 Department of Ocean Sciences, Tokyo University of Marine Science and Technology, Tokyo, Japan; 6 LEGOS, Université de Toulouse, CNRS, IRD, CNES, UPS, Toulouse, France; 7 Department of Geosciences, Princeton University, Princeton, NJ USA; 8 Univ Brest, CNRS, IRD, Ifremer, LEMAR, F-29280 Plouzane, France; 9 Woods Hole Oceanographic Institution, Falmouth, MA USA; 10 Department of Biological Sciences, University of Southern California, Los Angeles, CA USA; 11 Earth Sciences & Ocean Process Analysis Laboratory, University of New Hampshire, Durham, NH USA

**Keywords:** Marine chemistry, Ocean sciences

## Abstract

Dissolved organic phosphorus (DOP) concentration distributions in the global surface ocean inform our understanding of marine biogeochemical processes such as nitrogen fixation and primary production. The spatial distribution of DOP concentrations in the surface ocean reflect production by primary producers and consumption as an organic nutrient by phytoplankton including diazotrophs and other microbes, as well as other loss processes such as photolysis. Compared to dissolved organic carbon and nitrogen, however, relatively few marine DOP concentration measurements have been made, largely due to the lack of automated analysis techniques. Here we present a database of marine DOP concentration measurements (DOPv2021) that includes new (n = 730) and previously published (n = 3140) observations made over the last ~30 years (1990–2021), including 1751 observations in the upper 50 m. This dataset encompasses observations from all major ocean basins including the poorly represented Indian, South Pacific, and Southern Oceans and provides insight into spatial distributions of DOP in the ocean. It is also valuable for researchers who work on marine primary production and nitrogen fixation.

## Background & summary

Identifying and quantifying sources of nutrients fueling phytoplankton growth, especially in “ocean deserts”, where inorganic nutrient concentrations in sunlit surface waters are typically at or below detection limits, is important for understanding marine biogeochemical cycles. Given the scarcity of inorganic nutrients like nitrate (NO_3_^−^) and phosphate (PO_4_^3−^) in surface waters, considerable effort has gone into evaluating the potential for organic nitrogen (N) and phosphorus (P) compounds to support carbon and di-nitrogen (N2) fixation^1–5^. Unlike NO_3_^−^ and PO_4_^3−^, organic nutrients include a range of molecules that differ in structure and size, which makes them challenging to quantify. Dissolved organic phosphorus (DOP), for example, is operationally defined as any organic molecule containing at least one P atom that passes through a filter of a given pore size (often 0.2 to 0.7 µm)^6–8^. DOP concentrations ([DOP]) are measured indirectly, with [DOP] determined as the difference between measurements of total dissolved phosphorus ([TDP]) and soluble reactive P concentrations ([SRP], which is ~[PO_4_^3−^]):

1 $$\left[{\rm{DOP}}\right]=\left[{\rm{TDP}}\right]-\left[{\rm{SRP}}\right]$$

Quantitatively, the importance of DOP is highlighted by its dominance in the surface waters of oligotrophic gyres, where it accounts for up to ~80% of the total P pool^6,9^. Like dissolved organic carbon and nitrogen (DOC and DON, respectively), the principal source of marine DOP is primary production in surface waters^10^. DOP has also been found to be bioavailable to phytoplankton, thus supporting primary production and N_2_ fixation, especially when PO_4_^3−^ is scarce^1–3,11–16^. Many phytoplankton, notably *Trichodesmium* spp., *Thalassiosira* spp., *Synechococcus* spp., and *Emiliania huxleyi*, have been shown to utilize some portion of the DOP pool, for example by biosynthesis of extracellular alkaline phosphatase metalloenzymes under conditions of low [PO_4_^3−^]^1,14,17–20^. More generally, estimates from a global ocean circulation-biogeochemistry model, the Biogeochemical Elemental Cycling (BEC) model, suggest that global marine net primary productivity (NPP) and N_2_ fixation rates are ~8% and ~33% higher, respectively, when DOP is included as an assimilative P source^4^. Thus, DOP appears to play a significant role supporting biogeochemical cycling in the upper ocean.

**Fig. 1:**
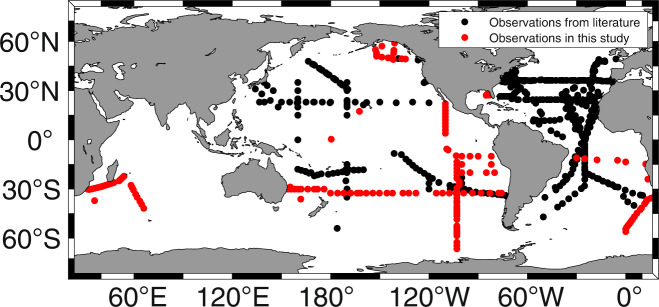
Global distribution of [DOP] observations in the DOPv2021 database. Black dots represent previously reported data and red dots represent new observations made as part of this study.

**Fig. 2:**
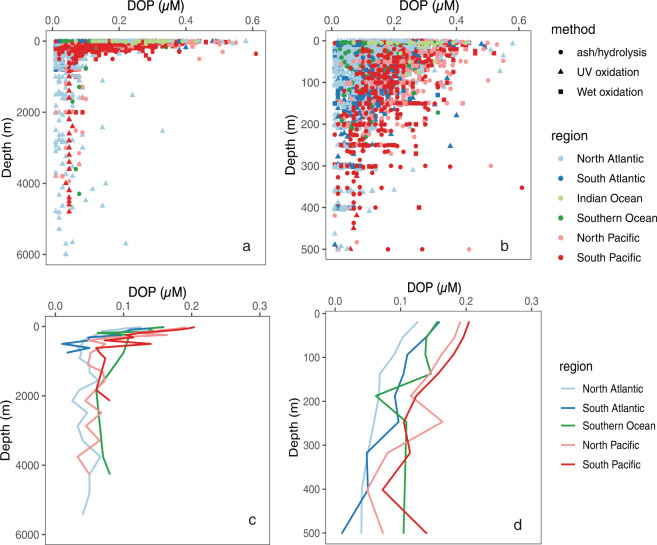
Profiles of [DOP] in the ocean. (**a**) Observations between 0–6000 m, (**b**) observations between 0–500 m, (**c**) mean [DOP] between 0–6000 m in different ocean basins, and (**d**) mean [DOP] between 0–500 m in different ocean basins. Mean [DOP] depth profiles were calculated after binning data into the OCIM2 grid^37,38^. Note that the deepest depth level in the OCIM2 grid is 5500 m. We did not include mean [DOP] profiles of the Indian Ocean in figures c and d due to limited observations from the Indian Ocean. Symbol colors represent ocean basin and symbol shapes in panels a and b represent [DOP] analytical method.

While phytoplankton both produce and consume DOP, observations at Station A Long-term Oligotrophic Habitat Assessment (ALOHA) and the Bermuda-Atlantic Time-series Study (BATS) site have shown that [DOP] is not uniform in the surface ocean, with higher concentrations at Station ALOHA in the North Pacific than at the BATS site in the Sargasso Sea in the North Atlantic (~0.2 µM vs. ~0.06 µM)^6,9,21,22^. Despite the importance of DOP for fueling primary productivity and N_2_ fixation, the spatial distribution of [DOP] and its variability in the ocean is still poorly resolved due to sparse measurements largely focused in the North Atlantic (Fig. 1). In particular, there are few published [DOP] measurements from the South Pacific, Indian Ocean and Southern Ocean. Prior efforts to compile marine [DOP] measurements, specifically, the Global Open Ocean DOP (GOOD) database^8^, were based on measurements made between 1932 and 1994, with 33% of the observations made prior to 1965 before the wet oxidation method (using potassium persulfate)^23^ and UV oxidation method^24^ were published, and 67% of the observations were made prior to 1980, before the publication of the ash/hydrolysis method^25^, which has been found to recover a higher fraction of some model DOP compounds than wet oxidation^26^. However, the [DOP] measurements in the GOOD database do not specify the method employed to measure [DOP], and many [DOP] measurements (2352 observations) reported in the GOOD database exceed 2 µM in open ocean surface waters, which is inconsistent with our current (i.e., < 30 years old) understanding of marine DOP (Fig. 2). Although the GOOD database has advanced our understanding of marine DOP^20^, the shortcomings described above limit its utility. As DOP observations have accumulated over the last 30 years, an updated, open access marine [DOP] database of analyses made using modern measurement techniques^23–27^ with corresponding metadata is required to facilitate marine biogeochemical research.

Here, we present a new DOP database (DOPv2021) with [DOP] measurements from more than 42 cruises undertaken over the past 30 years (1990–2021) (Supplementary Table 1), which are distinct from the data reported in the GOOD database, where corresponding methodological details are not reported. DOPv2021 includes 3870 observations of [DOP] with metadata for the majority of samples, including cruise name, sample location and depth, sampling date, analytical methods, corresponding hydrographic and inorganic nutrient concentration data, and reference information. Supplementary Table 1 indicates the source of previously published DOP data, as well as which measurements are new to this study.

## Methods

The DOPv2021 database includes previously reported [DOP] measurements as well as new measurements from the WebbPacific2007, CoFeMUG (KN192-05; GEOTRACES GAc01), ETSP2010, ETSP2011, Gulf of Alaska 2013 (GOA2013), GO-SHIP P18-2016, GO-SHIP P06-2017, Gulf of Mexico 2019 (GOM2019), SCALE 2019, and GEOTRACES-SWINGS (MD229; GEOTRACES GS02) cruises. Previously reported [DOP] measurements published between 1990 and 2021 are also included in the DOPv2021 database. A criterion for including previously published [DOP] data was analysis by one of the three modern methods (wet oxidation^23,27^, ash/hydrolysis^25,26^, and UV oxidation^24^). The database includes 730 new observations and 3140 observations from published literature. These new and previously reported [DOP] data were merged into one data file in the comma-separated format (i.e., a .csv file). Other non-proprietary community-established data formats include netcdf that can be downloaded directly from the BCO-DMO website (https://www.bco-dmo.org/dataset/855139/data).

Figure 1 shows where [DOP] samples included in the DOPv2021 database were collected. Supplementary Table 1 summarizes the number of [DOP] observations from each cruise, the cruise year, the analytical method employed to measure [DOP], and the data sources. Any negative [DOP] values are reported as “BDL” for “below detection limit” in the data file. These “BDL” [DOP] values are found in samples from the Southern Ocean portion of the GO-SHIP P18-2016 line (n = 35) and we do not include these “BDL” values in our data analysis. Although the focus of the database is [DOP] measurements, we also gathered salinity, temperature, nitrate + nitrite concentrations, and [SRP] along with the [DOP] measurements when these data were available. Supplementary Table 2 indicates whether salinity, temperature, nitrate + nitrite concentration, and/or [SRP] data associated with the [DOP] measurements are available for each cruise, along with the source of these data. Cruise information is also included in Supplementary Table 2 when available. Missing values in the DOPv2021 database are reported as “nd” for “no data”.

## Data Records

The DOPv2021 database and associated validation data are archived in the United States National Science Foundation-funded Biological and Chemical Oceanography Data Management Office (BCO-DMO) and can be accessed at both the BCO-DMO website (https://www.bco-dmo.org/dataset/855139) and the Woods Hole Open Access Server (10.26008/1912/bco-dmo.855139.3)^30^. Data associated with the quality of TDP measurements can be accessed under the supplemental file tab on the BCO-DMO web page. The DOPv2021 database includes the following information for each record:

EXPOCODE: the code defines a standard nomenclature for cruise labels of a

research vessel

Cruise: cruise name

LATITUDE (°N): latitude N (−90 to 90)

LONGITUDE (°E):longitude E (−180 to 180)

depth (m): sampling depth

date (yyyymmdd):sampling date

station ID: station number

bottle ID: Niskin bottle identifier or underway sample identifier

Temperature (°C): sample temperature

Salinity: sample salinity

NO3 + NO2 (µM): nitrate + nitrite concentration

SRP (µM): soluble reactive phosphorus concentration

DOP (µM): dissolved organic phosphorus concentration

DOP_flag:quality flag for DOP using WOCE bottle code

region: ocean basin sampled

method: analytical method employed to determine DOP concentration

reference: data source reference

## Technical Validation

### DOP concentration measurements

Although details vary, the protocol to measure the [DOP] of a sample included in this database can be simplified to four steps:

1. Determine the [SRP]
2. Oxidize the DOP of a sample to SRP
3. Determine the [TDP]
4. Take the difference of the [SRP] from the [TDP] in a sample (Eq. 1)

The three modern methods employed to measure [DOP] differ mainly in the oxidation approach (Step 2). There are three oxidation approaches used for the samples reported in the DOPv2021 database: wet oxidation, UV oxidation, and ash/hydrolysis. Wet oxidation is a chemical oxidation method that involves the addition of potassium persulfate to a seawater sample and the subsequent heating of the sample to 120 °C for 30 minutes to convert DOP to SRP^23,28^. UV oxidation is a photochemical oxidation method using UV radiation to convert DOP to SRP^24^ often used at the Hawaii Ocean Time-series (HOT) site^29^. We employed the ash/hydrolysis method^25,26^ for the new [DOP] measurements in this study. Seawater samples were filtered using polyesthersulfone (PES) filters with a nominal 0.2 µm pore size or combusted Whatman glass fiber filters (GF/F) with a nominal 0.7 µm pore size. Filtrates were collected in HDPE bottles and immediately stored at −20 °C until analysis in the lab. For samples collected >1 year before [TDP] analysis, the sample pH was reduced to < 2 by adding ~150 µL 6 M ACS-grade HCl (Fisher Chemical, A144C-212) to the sample bottle and placing the bottle in a reciprocal shaker overnight in order to solubilize PO_4_^3−^ adsorbed to the bottle wall, see below. Then, 6 mL of the sample was added to an acid washed, 500 °C combusted glass vial, and 0.6 mL of 4.3 M NaCl/0.3 M MgSO4 solution was added to the sample. Subsequently, vials were put into a drying oven at 70 °C until dry (often 4 to 5 days). Then, each vial was covered with aluminum foil and transferred to a muffle oven to bake at 130 °C for 3 hours and then at 500 °C for 4.5 hours. Afterwards, 1.8 mL 0.75 M ACS-grade HCl (Fisher Chemical, A144C-212) was added to each vial that was then capped tightly with a Teflon-lined cap, then heated at 80 °C for 20 min to hydrolyze the polyphosphate left after ashing. After heating, 4.2 mL ultrapure water (18.2 MΩ·cm^−1^) was added to each vial and heated at 80 °C for 10 min to dissolve all remaining solids. We assumed quantitative conversion of DOP to SRP and the resulting [SRP] was measured by the colorimetric phosphomolybdate-blue method^26,27^ on a Shimadzu 1800 UV-vis spectrophotometer at 880 nm to determine [TDP].

We also collected [DOP] measurements from the literature if one of the three modern methods was used to measure [DOP] (i.e., wet oxidation, ash/hydrolysis, or UV oxidation). However, no inter-comparison of [DOP] data generated using the different methods was conducted in this study.

### Quality of DOP concentration measurements

The quality of [DOP] measurements can be evaluated in three ways: 1) measuring the recovery of model DOP compounds, 2) quantifying the analytical blank, and 3) evaluating the reproducibility of the [TDP] of the same sample over time.

To evaluate the effectiveness of the ash/hydrolysis method used in this study to recover the DOP in a sample, we included two model compounds with known concentrations (0.5–2.0 µM), adenosine triphosphate (ATP) and glyphosate (GLY) in each batch of oxidized samples. These two model compounds were processed alongside seawater DOP samples, providing information on the recovery of DOP by the analytical method. The recovery of ATP and GLY in our study was 92 ± 6% (n = 27) and 91 ± 7% (n = 31), respectively, consistent with the reported recovery of model compounds by the ash/hydrolysis method in Monaghan & Ruttenberg, 1999. While we do not know whether the previously reported [DOP] measurements in the DOPv2021 dataset included the analysis of model compounds, prior work has shown that the wet oxidation, ash/hydrolysis, and UV oxidation [TDP] methods recover roughly >90% of model DOP compounds^26,30^, implying similar levels of DOP recovery in the DOPv2021 dataset, regardless of analytical method employed.

In order to quantitatively compare the contribution of DOP contamination from the methodological procedures relative to the DOP in the sample, we prepared ultra-pure (“Milli-Q”) water in the same way as the samples and evaluated potential DOP contributions from reagents and sample handing during the sample analysis. The mean (±1 S.D.) [DOP] of the Milli-Q water blank was 0.00 ± 0.03 µM (n = 34), indicating no significant contribution of DOP from reagents and sample analysis and handling, and that the [DOP] reported should only reflect the DOP within a seawater sample, and not from any analytical contamination.

**Fig. 3:**
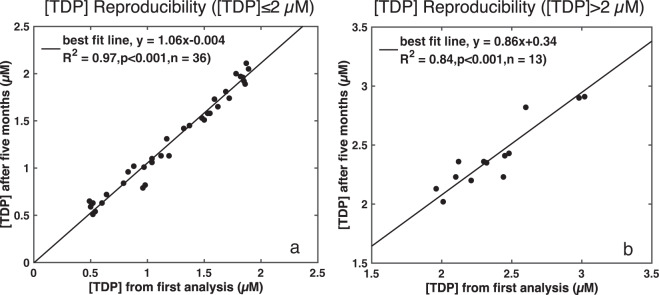
Reproducibility of [TDP] measurements. The second [TDP] analysis took place five months after the first analysis. (**a**) Reproducibility of samples with [TDP] ≤ 2 µM (n = 36), and (**b**) reproducibility of samples with [TDP] >2 µM (n = 13). The black line represents the best fit line determined from a Type II regression model.

To evaluate the precision of our [DOP] measurements, we also included 0.2 µm filtered surface water from the oligotrophic Gulf of Mexico to check the reproducibility of DOP concentrations over time. The mean (±1 S.D.) [DOP] in Gulf of Mexico surface seawater was 0.12 (±0.04) µM (n = 23). The [SRP] in this Gulf of Mexico sample was below the detection limit (50 nM), indicating that in this sample [TDP] ~ [DOP]. In order to test the precision of the [TDP] measurement over a wider range of [TDP], we re-analyzed samples with different [TDP]. We found higher reproducibility (R^2^ = 0.97, n = 36) for samples with ≤ 2 µM [TDP] than >2 µM [TDP] (R^2^ = 0.84, n = 13) in samples re-analyzed five months after the original [TDP] analysis (Fig. 3). The poorer reproducibility of samples with elevated [TDP] corresponds to higher [SRP] in the same samples, contributing to greater uncertainty in the [DOP] determination. Samples with [TDP] >2 µM are common from the deep ocean and regions where upwelling brings SRP-replete deep water to the surface, such as the Southern Ocean and coastal regions.

Importantly, we estimate that if TDP samples have been stored at −20 °C for >1 year, detectable levels of SRP may be lost to bottle walls, resulting in the underestimation of [TDP] measurements due to under-recovery of SRP (Table 1), although this result is only based on two samples with high [SRP]. We suggest that this is due to adsorption of detectable levels of PO_4_^3−^ to the bottle wall after ~1 year, but further study of the mechanism is needed. As a result, the [DOP] of a sample may be underestimated and even negative if the [SRP] was measured within the first year of sample collection, but [TDP] analysis occurred later. To address this, after thawing, we acidified TDP samples frozen for >1 year with ACS-grade HCl to pH < 2 to release the PO_4_^3−^ adsorbed to the bottle wall back into the seawater. Comparison of the [SRP] of two samples measured at sea (ETSP2010) with the [SRP] of the same samples stored at −20 °C for seven years, before and after acidification, are reported in Table 1. The [SRP] of un-acidified samples stored for seven years was 72–91% of the [SRP] measured at sea while the [SRP] of the sample stored for seven years after acidification was 95–98% of the [SRP] measured at sea. Effectively complete recovery of SRP, and thus TDP, was achieved by acidifying samples to pH < 2 and leaving the acidified samples on a reciprocal shaker overnight before measuring [TDP] (Table 1). For all [DOP] reported in this study (i.e., red symbols in Fig. 1), [SRP] was measured within 1 year of sample collection.

**Table 1:** The effect of storing samples at –20 °C for seven years on measured sample [SRP] with and without sample acidification.

Sample	[SRP] (µM)			
	Measured at sea	Frozen 7 years, unacidified	Frozen 7 years, 30 min acidified to pH < 2	Frozen 7 years, overnight acidified to pH < 2
ETSP-2500 m	2.91	2.11	2.86	2.86
ETSP-2000 m	3.04	2.79	2.89	2.91

### Validation and sources of other measurements

Data sources and details of temperature, salinity, SRP concentration, and nitrate + nitrite concentration data associated with each cruise can be found in Supplementary Table 2 and references therein.

## Usage Notes

### Summary of [DOP] observations

**Fig. 4:**
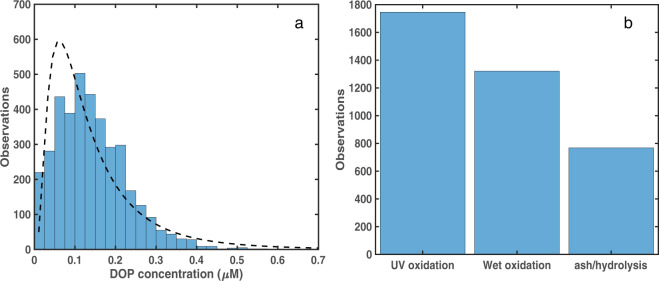
Histograms showing (**a**) [DOP] and (**b**) the number of [DOP] measurements made using different analytical methods. The dashed line in panel a depicts the fit of the data to a log-normal distribution.

The [DOP] distribution in the DOPv2021 database follows a log-normal distribution (Fig. 4a), with most observations falling between 0.10 and 0.20 µM (n = 1726) and 179 observations with [DOP] >0.30 µM (maximum = 0.61 µM) (Fig. 4a). There are 1746 [DOP] observations in the DOPv2021 database made using the UV oxidation method, 1321 [DOP] observations made using the wet oxidation method, and 768 [DOP] observations made using the ash/hydrolysis method (Fig. 4b). We note that samples measured using the UV oxidation method are primarily from the Atlantic Ocean, with wet oxidation or ash/hydrolysis methods mainly used in Pacific and Indian Ocean samples (Fig. 2) (Supplementary Table 1), potentially adding analytical bias to the global [DOP] distributions.

The DOPv2021 database includes measurements from the surface to 6000 m (Fig. 2). Of these, 1751 observations are from the upper 50 m, 3234 observations are from the upper 200 m, and 132 observations are from >1000 m (Fig. 2), reflecting interest in DOP’s role in upper ocean biogeochemistry. The mean [DOP] in the upper 50 m of the global ocean was 0.17 ± 0.08 µM. Upper ocean [DOP] varies more between ocean basins than deep ocean [DOP] (Fig. 2). The deep ocean (>1000 m) [DOP] reflects the refractory portion of the DOP pool, with a mean deep ocean [DOP] in the DOPv2021 database of 0.05 ± 0.05 µM (n = 132), consistent with the deep ocean [DOP] reported at Station ALOHA^22^ and in Letscher & Moore, 2015^4^ (Table 2). In contrast, the mean deep ocean [DOP] and associated standard deviation in the GOOD database is much higher, 0.12 ± 0.2 µM (Table 2). As has been discussed previously^22^, [SRP] >2 µM limits the precision of [DOP] estimates in the deep ocean, especially when the [SRP] approaches the [TDP] and the difference between [SRP] and [TDP] is small. We find three outliers among deep ocean [DOP] observations based on Chauvenet’s criterion, which is applied to normally distributed datasets and rejects data whose probability of deviation from the mean is <1/(2n) (where “n” is sample size)^31^. The mean deep ocean [DOP] does not change if these three outliers are excluded, but the standard deviation without these three outliers decreases to 0.03 µM. Additional [DOP] measurements from the deep ocean will better constrain the rates and locations of heterotrophic and abiotic DOP sink processes.

**Table 2:** Deep ocean [DOP] reported at Station ALOHA, from the GOOD DOP database, in Letscher & Moore, 2015, and from the DOPv2021 database.

Data Source	Location	Depth range (m)	Mean (±1 S.D.) DOP (µM)
Station ALOHA^22^	North Pacific	900 - 4800	0.049 ± 0.004
GOOD DOP^8^	Global ocean	>1000	0.16 ± 0.2
Letscher & Moore, 2015^4^	Global ocean	>1000	0.03 ± 0.02
DOPv2021	Global ocean	>1000	0.05 ± 0.03

**Fig. 5:**
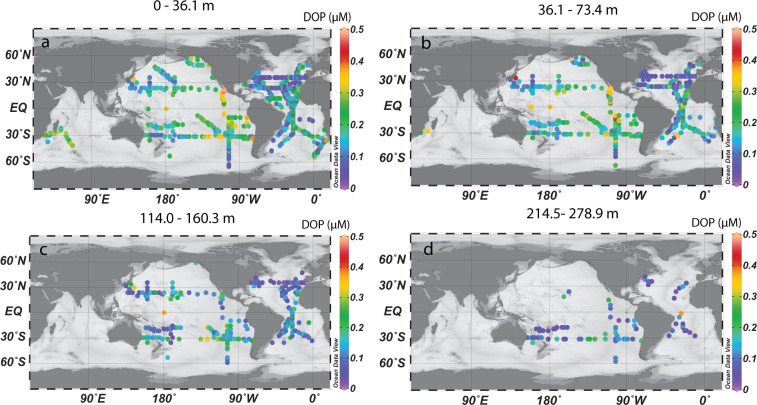
Examples of [DOP] distributions in the DOPv2021 database binned onto a 2° × 2° OCIM2 grid. [DOP] distributions are shown for the following depth bins include: (**a**) surface (0 to 36.1 m); (**b**) 36.1 to 73.4 m; (**c**) 114.0 to 160.3 m; and (**d**) 214.5 to 278.9 m. More information about the OCIM2 grid can be found in DeVries & Primeau, 2011 and John *et al*., 2020^37,38^.

**Fig. 6:**
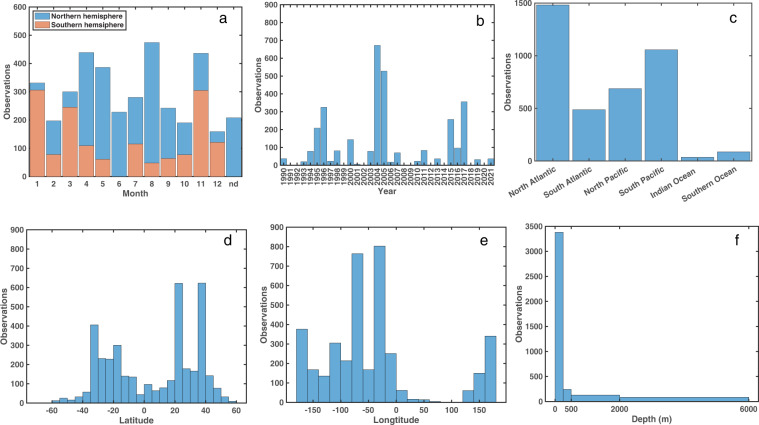
Temporal and spatial distributions of [DOP] observations. (**a**) The number of observations as a function of sampling month (“nd” are samples for which the sampling month is not reported), with blue bars representing observations from the northern hemisphere and orange bars representing observations from the southern hemisphere; (**b**) observations as a function of sampling year; (**c**) observations from each ocean basin; (**d**) observations as a function of latitude; (**e**) observations as a function of longitude; and (**f**) observations as a function of sampling depth.

The DOPv2021 database includes [DOP] measurements from all major ocean basins (Supplementary Table 1) (Fig. 1). However, these observations are not evenly distributed (Figs. 1,5 and 6). In particular, the Indian and Southern Oceans remain under-sampled (Figs. 5,6). Sparse [DOP] observations from the Southern Ocean hinder our understanding of the spatial and temporal variability of [DOP] in the global surface ocean, as well as of the fertility of the adjacent low-latitude Southern hemisphere gyres. For example, we might expect low [DOP] in recently upwelled deep waters, with higher [DOP] resulting from high rates of new production in the Southern Ocean spring and summer, as has been observed for DOC and DON in regions impacted by upwelling^32–34^. Since [DOP] observations in the DOPv2021 database are concentrated in the mid-latitudes (Fig. 6), where oligotrophic gyres with low [SRP] result in DOP being a significant assimilative P source^2,3,13^, we cannot evaluate the role of productivity in the Southern Ocean as a source of DOP to low-latitude gyres, although we expect it to be significant^35^. The lack of [DOP] measurements from the Indian Ocean highlights another region that requires further investigation.

### Temporal and spatial distribution of [DOP] observations

[DOP] measurements in the DOPv2021 database include observations from every month, although observations are concentrated in the spring and fall (Fig. 6a). The small but detectable seasonal changes in [DOP] observed at the BATS site^6^ raise the possibility of seasonal bias in the DOPv2021 database, and underscore the need for additional [DOP] measurements from samples collected during the summer and winter.

There are several general features of the global ocean [DOP] distributions. First, [DOP] decreases with increasing depth (Figs. 2,5). However, [DOP] in the upper 200 m varies significantly among ocean basins (Figs. 2,5). Additionally, [DOP] gradients occur in the upper 50 m across ocean basins (Fig. 5). For example, in the Pacific Ocean, upper 50 m [DOP] is higher on the eastern side of the basin (mean ± 1 S.D. of 0.24 ± 0.08 µM for observations east of 160°W, n = 319), and lower in the west (mean ± 1 S.D. of 0.17 ± 0.06 µM for observations west of 160°W, n = 418) (Fig. 5). Additionally, upper 50 m [DOP] is relatively high between 20°S and 20°N (mean ± 1 S.D. of 0.20 ± 0.08 µM, n = 391) and lower in the center of the oligotrophic gyres between 20° and 40° north or south (mean ± 1 S.D. of 0.16 ± 0.08 µM, n = 1097) (Fig. 5), reflecting regions of net production and consumption, respectively^16^.

### Database summary

This DOPv2021 database reports global [DOP] observations made using modern methods and includes corresponding metadata such as data sources and methods. This dataset can be used to explore [DOP] distributions at basin and global scales, as well as the relationship between [DOP] and other parameters. This dataset also functions as a [DOP] field that can be assimilated into ocean biogeochemical models. Researchers should, however, be aware of the potential biases inherent to the dataset, including: 1) seasonal bias with higher coverage in spring and fall; 2) poor coverage of the Southern and Indian Oceans; 3) methodological bias, with samples preferentially analyzed using the UV oxidation method in the Atlantic Ocean; and, 4) limited deep ocean [DOP] data. Finally, new [DOP] measurements can be added to the database by contacting the corresponding author. Updates to the database are reflected in updated version numbers of the database as well as in corresponding metadata recorded at the BCO-DMO site.

## Supplementary information

Supplementary Information

Supplementary Table 1

Supplementary Table 2

## Data Availability

No code was used to generate this dataset. The codes to reproduce Figs. 1,2,3,4, and 6 shown in the article are publicly available in the GitHub (https://github.com/zliangocean/DOPv2021). Figure 5 was plotted by using Ocean Data View^36^ software (odv.awi.de).
