# Supplementary material for: A global ocean dissolved organic phosphorus concentration database (DOPv2021)
Source: Sci Data. 2022 Dec 16;9:772. doi: 10.1038/s41597-022-01873-7 (PMC9758185; doi:10.1038/s41597-022-01873-7)
Supplement: Supplementary file 1 — Supplementary Information [file 41597_2022_1873_MOESM1_ESM.pdf]

A global ocean dissolved organic phosphorus concentration database (DOPv2021)

Zhou Liang[1], Kelly McCabe[2], Sarah E. Fawcett[3,4], Heather J. Forrer[1], Fuminori Hashihama[5], Catherine Jeandel[6], Dario Marconi[7], Hélène Planquette[8], Mak A. Saito[9], Jill A. Sohm[10], Rachel K. Thomas[1], Robert T. Letscher[11], Angela N. Knapp[1]

[1]Department of Earth, Ocean, and Atmospheric Science, Florida State University, Tallahassee, FL, USA

[2]Copperative Institute for Great Lakes Research (CIGLR), School for Environment and Sustainability, University of Michigan, Ann Arbor, MI, USA

[3]Department of Oceanography, Faculty of Science, University of Cape Town, Cape Town, South Africa

[4]Marine and Antarctic Research centre for Innovation and Sustainability (MARIS), University of Cape Town, Cape Town, South Africa

[5]Department of Ocean Sciences, Tokyo University of Marine Science and Technology, Tokyo, Japan

[6]LEGOS, Université de Toulouse, CNRS, IRD, CNES, UPS, Toulouse, France

[7]Department of Geosciences, Princeton University, Princeton, NJ, USA

[8]CNRS, IRD, Ifremer, LEMAR, Univ Brest, Plouzané, France

[9]Woods Hole Oceanographic Institution, Woods Hole, MA, USA

[10]Department of Biological Sciences, University of Southern California, Los Angeles, CA, USA

[11]Earth Sciences & Ocean Process Analysis Laboratory, University of New Hampshire, Durham, NH, USA

Corresponding author: Zhou Liang (zl18c@fsu.edu)

Supplementary information

Supplementary Table 1. Summary of data sources for [DOP] measurements included in the DOPv2021 database.

Supplementary Table 2. Data source and cruise information for the temperature (T), salinity (S), nitrate + nitrite (NO3+NO2) concentrations, and [SRP] associated with the [DOP] measurements. NA indicates “not available”

References

1. Ridal, J. J. & Moore, R. M. Dissolved organic phosphorus concentrations in the northeast subarctic Pacific Ocean. *Limnol. Oceanogr.* **37**, 1067–1075 (1992).

2. Hopkinson, C. S. & Vallino, J. J. Efficient export of carbon to the deep ocean through dissolved organic matter. *Nature* **433**, 142–145 (2005).

3. Vidal, M., Duarte, C. M. & Agusti, S. Dissolved organic nitrogen and phosphorus pools and fluxes in the central Atlantic Ocean. *Limnol. Oceanogr.* **44**, 106–115 (1999).

4. Loh, A. N. & Bauer, J. E. Distribution, partitioning and fluxes of dissolved and particulate organic C, N and P in the eastern North Pacific and Southern Oceans. *Deep Sea Res. Part Oceanogr. Res. Pap.* **47**, 2287–2316 (2000).

5. Cavender-Bares, K. K., Karl, D. M. & Chisholm, S. W. Nutrient gradients in the western North Atlantic Ocean: relationship to microbial community structure and comparison to patterns in the Pacific Ocean. *Deep Sea Res. Part Oceanogr. Res. Pap.* **48**, 2373–2395 (2001).

6. Mather, R. L. *et al.* Phosphorus cycling in the North and South Atlantic Ocean subtropical gyres. *Nat. Geosci.* **1**, 439–443 (2008).

7. Suzumura, M. & Ingall, E. D. Distribution and dynamics of various forms of phosphorus in seawater: insights from field observations in the Pacific Ocean and a laboratory experiment. *Deep Sea Res. Part Oceanogr. Res. Pap.* **51**, 1113–1130 (2004).

8. Sohm, J. A. & Capone, D. G. Phosphorus dynamics of the tropical and subtropical north Atlantic: Trichodesmium spp. versus bulk plankton. *Mar. Ecol. Prog. Ser.* **317**, 21–28 (2006).

9. Raimbault, P., Garcia, N. & Cerutti, F. Distribution of inorganic and organic nutrients in the South Pacific Ocean − evidence for long-term accumulation of organic matter in nitrogen-depleted waters. *Biogeosciences* **5**, 281–298 (2008).

10. Yoshimura, T. *et al.* Distributions of particulate and dissolved organic and inorganic phosphorus in North Pacific surface waters. *Mar. Chem.* **103**, 112–121 (2007).

11. Reinthaler, T., Sintes, E. & Herndl, G. J. Dissolved organic matter and bacterial production and respiration in the sea-surface microlayer of the open Atlantic and the western Mediterranean Sea. *Limnol. Oceanogr.* **53**, 122–136 (2008).

12. Lomas, M. W. *et al.* Sargasso Sea phosphorus biogeochemistry: an important role for dissolved organic phosphorus (DOP). *Biogeosciences* **7**, 695–710 (2010).

13. Hashihama, F. *et al.* Biogeochemical controls of particulate phosphorus distribution across the oligotrophic subtropical Pacific Ocean. *Glob. Biogeochem. Cycles* **34**, e2020GB006669 (2020).

14. Moutin, T. *et al.* Nutrient availability and the ultimate control of the biological carbon pump in the western tropical South Pacific Ocean. *Biogeosciences* **15**, 2961–2989 (2018).

15. Foreman, R. K., Björkman, K. M., Carlson, C. A., Opalk, K. & Karl, D. M. Improved ultraviolet photo-oxidation system yields estimates for deep-sea dissolved organic nitrogen and phosphorus. *Limnol. Oceanogr. Methods* **17**, 277–291 (2019).
